# Test implementation of a school-oriented drug prevention program “Study without Drugs”: pre- and post-testing for effectiveness

**DOI:** 10.1186/1471-2458-14-590

**Published:** 2014-06-11

**Authors:** Fariel Ishaak, Nanne Karel de Vries, Kees van der Wolf

**Affiliations:** 1 Faculty of Social Science, Anton de Kom University of Suriname, Leysweg 86, Suriname; 2 Department of Health Promotion, CAPHRI School for Public Health and Primary Care, Maastricht University, P.O. Box 616, 6200 MD Maastricht, The Netherlands; 3 Faculty of Social and Behavioural Science University van Amsterdam, P.O. Box 19268, 1000 GG Amsterdam, The Netherlands

**Keywords:** Adolescents, School prevention program, Drugs

## Abstract

**Background:**

In this article, the test implementation of a school-oriented drug prevention program “Study without Drugs” is discussed. The aims of this study were to determine the results of the process evaluation and to determine whether the proposed school-oriented drug prevention program during a pilot project was effective for the participating pupils.

**Methods:**

Sixty second-grade pupils at a junior high school in Paramaribo, Suriname participated in the test implementation. They were divided into two classes. For the process evaluation the students completed a structured questionnaire focusing on content and teaching method after every lesson. Lessons were qualified with a score from 0–10. The process was also evaluated by the teachers through structured interviews. Attention was paid to reach, dose delivered, dose received, fidelity, connection, achieved effects/observed behaviors, areas for improvement, and lesson strengths. The effect evaluation was conducted by using the General Liniair Model (repeated measure). The research (-design) was a pre-experimental design with pre-and post-test.

**Results:**

No class or sex differences were detected among the pupils with regard to the assessment of content, methodology, and qualification of the lessons. Post-testing showed that participating pupils obtained an increased knowledge of drugs, their drug-resisting skills were enhanced, and behavior determinants (attitude, subjective norm, self-efficacy, and intention) became more negative towards drugs.

**Conclusions:**

From the results of the test implementation can be cautiously concluded that the program “Study without Drugs” may yield positive results when applied in schools). Thus, this pilot program can be considered a step towards the development and implementation of an evidence-based school-oriented program for pupils in Suriname.

## Background

The test implementation of a school-oriented drug prevention program “Study without Drugs” was conducted in Suriname. Suriname is located in the northeast of South America and has an area of l163,820 km^2^. The population consists of ± 600,000 people and is very heterogeneous in composition. After a ± 300-year period of colonization, first by England and then the Kingdom of the Netherlands, Suriname became an independent republic on November 25, 1975. On November 25, 1980 a coup d’ etat took place, led by Desi Bouterse. During the period 1980-1987 Suriname was more often associated with drug trafficking. Democracy did his re-appearance in 1987. Studies suggest that there are no previously conducted evidence-based drug prevention programs developed and implemented in schools. “Study without Drugs” is the first school-based drug prevention program which during its development possible criteria of other effective school-based drug prevention programs have been integrated as much as possible. This program can serve as a first step towards the further development of evidence-based school programs in Suriname.

School plays an important role with regard to the presentation of interventions. Adolescents spend a great deal of time in school, and general education and the teaching of life skills are central in educational environments. Drug prevention programs in schools focus on teaching and/or improving skills and knowledge about drugs and the behavior determinants of drug use. Teachers can help improve and maintain the wellbeing of pupils by detecting and preventing drug use [1]. At school, information on drugs can/must be offered because defensible pupils perform better [2-5]. Thus, it is important to identify effective factors when determining the impact of such programs. This is necessary to properly rate the value of the executed programs and to make choices for theoretical methods and practical strategies.

In addition to identifying effective factors, school-oriented prevention programs must be evidence based. A method or approach is evidence based when its function has been proved through scientific research [6,7].

The evidence should have been found largely through ‘hard’ research methods like experiments with randomly selected experimental and control groups. Van der Wolf and Van Beukering [8] indicated that evidence based means that the methods, programs, and approaches used have to be scientifically proven to be effective. An evidence-based approach promotes reflection on the effectiveness of the methods, approaches, and programs used. Influencing factors are brought into the picture and analyzed. One speaks of evidence-based drug prevention when the strategies, procedures, and methods used are carefully validated and standardized under research conditions and are found effective with regard to behavior changes in adolescents concerning drug use and drug attitudes [9].

A test implementation can be considered small-scale evaluation research. Evaluation research is defined as “a process for applying academic procedures when gathering reliable and valid evidence regarding the manner and the scale of which specific activities produce certain results and outcomes” [10].

Evaluation research measures the effects and outcomes of programs and suggests ways for refining these programs to obtain more favorable outcomes [11].

### Process evaluation and effect evaluation

Process evaluation can focus on various levels (content/organization) and target groups (intermediary and end target groups), and can be conducted by applying several research methods. Process evaluation is also used to determine whether the intervention on each target group was implemented uniformly. Process evaluation provides information about the reasons behind a program’s effectiveness or ineffectiveness. Whether or not the intervention has an influence is also measured, and the reasons behind its success or failure. Many interventions consist of several activities. Process evaluations can then be used to determine how each activity influences the outcome results separately. It is important to know what activities the most or the least contributed to this success. This type of evaluation will help explaining the relationship between specific intervention activities. Process evaluation can help to prove why the intervention failed. Examples of failure of interventions are: inappropriate intervention design, incomplete implementation and insufficient accessibility of the target audience, an intervention can be adjusted to make it yet successful, through a process analysis.

Thus, research is conducted on the conditions necessary for the effect. Furthermore, insight is given into possible improvements or revisions, for example, to improve the intervention design and methods for the future [12-14]. The following components are also discussed with regard to process evaluation: context, reach, dose delivered, dose received, fidelity, implementation, and recruitment [15]. Not all components need to be included in a process evaluation. Priority should be given to questions on the size, dose delivered, dose received, and consistency.

With effect evaluation, the effects on behavior are measured. Effect evaluation can focus, for instance, on changes in behavior determinants. It is not always possible to measure hard, precise, and objective outcomes with health-promotion interventions. For this reason, secondary effect measures are established and measured. The secondary measures are behavior determinants that may have been influenced during and because of the intervention. The idea is that change in determinants will lead to a change in behavior [16]. Thus, two questions can be asked: 1) has the program attained its short-term expectations and 2) has the program achieved the desired long-term results?

### Drug use among Surinamese adolescents

The Drug Demand Reduction Program (DDR) conducted a nationwide Rapid Situation Assessment in 2005 [17]. The assessment studied 480 participants aged 13 years and older. The results showed that the majority of smokers started smoking before they were 20 years old.

More than 50% of the youngsters (13-16 year) indicated that their fathers smoked. With relation to age, 60% of the male respondents had their first alcoholic drink before turning 20, as did 38% of the female respondents.

School surveys conducted in 2004 [18] and 2006 [19] by the DDR (by order of CICAD [Inter-American Drug Abuse Control Commission]) found alcohol, cigarettes, and marijuana to be the most commonly used drugs. Cigarettes and marijuana were predominantly used by male students aged between 13 and 15 years. Female students most commonly used alcohol for the first time when aged between 13 and 14 years. Most students who smoked were in the second year of high school and those who used alcohol were in their first year. On average, 30% of the respondents had access to illicit drugs. For detailed data on drug use among Surinamese adolescents, please see Tables 1 and 2.

**Table 1:** Prevalence of drug abuse alcohol and drug abuse in secondary schools survey, 2006

**Type of drug**	**Lifetime (percentage)**	**Last 12 months (percentage)**	**Last 30 days (percentage)**
Cigarettes	35.8	15.2	7.9
Alcohol	63.5	46.8	34.4
Tranquillizers	9.8	5.5	3.2
Stimulants	4.8	2.7	1.5
Solvents & Inhalants	7.3	3.4	2.1
Marijuana	6.8	4.1	2.3
Hashish	1.5	0	0
Hallucinogens	0.4	0	0
Heroin	0.5	0	0
Opium	0.2	0	0
Morphine	0.3	0	0
Cocaine HCL	0.6	0.2	0.1
Coca Pasta	0.7	0	0
Crack	0.6	0.3	0
Ecstasy	1.2	0.2	0.2
Other drugs	3.5	2	1
Any illicit drug	16.9	9	5.2

**Table 2:** First use age alcohol and drug abuse in secondary schools survey, 2006

**Type of drug**	**Mean**
Cigarettes	12.9
Alcohol	13.2
Tranquillizers	13
Stimulants	13.7
Solvents & Inhalants	11.9
Marijuana	15.2
Hashish	15.4
Other drugs	12.9
Any illicit drug	13.7

The DDR (2005) [20] also executed a small-scaled study among 125 adolescents aged between 12 and 16. The results of this research are as follows: 20% of respondents stated that they knew someone (or more than one person) that used drugs. The drugs users were, for instance, direct family members, friends, schoolmates, or strangers (e.g., people from the neighborhood and junkies). The most commonly used drugs were tobacco (29.6%), and marijuana and alcohol (25%).

Based on the available data, alcohol, tobacco, and marijuana are popular among youth in Suriname. Although not many young people use cocaine, they could easily point out people in their social environment who did. This leads to the assumption that cocaine is readily available among the Surinamese population.

Thus, while the level of drug use among youth in Suriname is not alarmingly high (yet), prevention measures must be implemented to ensure it does not escalate.

### Intervention mapping protocol

To interpret what has been recorded concerning evidence-based school-oriented drug prevention programs, and keeping in mind the assumption that the presented data from the executed school drug surveys and other (small-scale) studies represent just the tip of drug use, a school-oriented drug prevention program “Study without Drugs” was developed using intervention mapping (IM) protocol. “Study without Drugs” is a drugs demand-reduction program that has been developed for Surinamese adolescents at junior high level. The development of the drug prevention program was carried out using IM protocol, which consists of six steps. The added value of this protocol is that it lends itself to prepare and execute health-promotion activities. IM is based on theory, empirical evidence, and additional qualitative and quantitative research, and the involvement of stakeholders and actors is central. The six phases of the protocol enable health-promotion activities to be prepared and executed, taking into account situational and personal circumstances. In phase 6, an evaluation plan is made that focuses on effect and process evaluations of the program, and an evaluation model is developed [13]. The school-oriented drug prevention program developed in March–April 2010 consists of 10 activities, namely: seven lessons (e.g., Dutch, biology, physical education, drawing, religion, and social studies), one informative activity, and two evaluation procedures. The intervention was carried out from June 2010 until –July 2010 (see Table 3). In designing “Study without Drugs” attempts have been made to integrate many criteria which have been proven to be effective in school programs, such as: use of interactive methods; “Teaching of Skills” is central; training of persons who are going to offer the program is necessary; the research design includes a pre-and post-test; an integrated approach and position of the prevention program within the curriculum is desirable; peers as implementers ensure increased effectiveness; activities that fall outside the curriculum should be included; and there is an approach where the family (parents), the society or community or parts thereof are involved or have an input in the program [21-24].

**Table 3:** Overview program plan

**Act nr**	**Activity**	**Aim**	**Means/material**
1	Conduct pre-test regarding drugs (duration 30 minutes)	Measuring knowledge and attitude with regard to drugs	Questionnaire
2	Interpersonal information activities	Ask attention for the drugs issue and offer information/knowledge with regard to drugs prevention program	Folder, power-point presentation drugs in kind
	Duration: 2 hours		
3	Subject Biology	- Categorize mentioned drinks	Developed lesson plan, drugs info book, pictures
	Consequences of alcohol, tobacco, marijuana and cocaine	- State consequences and have discussion	
	Duration:40 minutes		
4	Subject Physical Education	- Educational conversation about ethics and mentioning 4 consequences of drugs use in sports	Developed lesson plan, pictures, for instance of steroids (in kind)
	Sports and drugs (steroids)	- Measures to protect against drugs use	
	Duration: 40 inutes		
5	Subject Dutch	- Put events in the right order and discuss them	Developed lesson plan, pictures, blackboard, and chalk
	Discussion about the consequences of swallowing cocaine pellets	- Indicate consequences of swallowing pellets	
	Duration: 80 minutes		
6	Subject Social Studies	- Put events in the right order	
	(Broadcast of play with anti drugs message)	- Arrive at a clear view on drugs and youngsters	
	Duration: 60 minutes	- Indicate causes and consequences that lead to drugs use among (school going) youngsters	
		- Think up protective measures	
7	Subject Dutch	- Write and present slogan	Developed lesson plan, drugs info book, pictures of, for instance, slogans, blackboard, and chalk
	Write an anti drugs slogan		
	Duration: 40 minutes		
8	Subject Drawing	- Design poster	Developed lesson plan, folders, poster, drawing material, chalk, drugs info book
	Posters with anti drugs message		
	Duration: 40 minutes		

## Methods

### Study participants

The two groups of respondents (all pupils from one junior high school) consisted of 60 pupils: 24 boys and 36 girls aged between 12 and 15 years, with an average age of 14 years. Class 2A had 31 pupils (12 boys and 19 girls) and 2B 29 pupils (12 boys and 17 girls). It was a select sample survey based on voluntary participation. School management and teachers committed themselves to carrying out the test implementation at their school. The group of teachers consisted of three women and two men (four teachers had been in service for less than 10 years). Two teachers were qualified to teach at senior high level, two at junior high level, and one had a different teaching qualification. Carrying out the surveys and interviews for both the process evaluation and the pre-and post-test were done by trained interviewers who were not known to the students nor the teaching staff.

### Process evaluation

The process evaluation by the pupils was done via a questionnaire (developed by the researcher) after each lesson. The objective of the process evaluation by the pupils was to record their opinion on the content of the lessons taught and the teaching method. The pupils also got the opportunity to express whether or not they enjoyed the lessons by qualifying the lesson with a number between 1 and 10. The pupils had a maximum of 30 minutes to fill in the questionnaire.

A structured interview was also conducted with the teachers after each lesson. The interview lasted 45–60 minutes and consisted of questions on the following aspects: reach, dose delivered, dose received, fidelity, subject connection, general remarks, lesson strengths, and areas for improvement. These interviews were recorded with a voice recorder and later transcribed into a computer.

The transcribed conversations were presented to the respondents for possible modification (comments). Then the data were coded and the transcripts analyzed.

### Effect evaluation

For the effect evaluation, a pre-test was conducted before the execution of the program, after two-and-a-half month followed by a post-test after the program. Each of the tests lasted 35 minutes. Measurements were taken on the basis of a one-group pre- and post-test design. These tests aimed to measure the pupils’ knowledge, skills, and the impact of behavior determinants before and after attending the prevention program.

### Measuring instruments

The questionnaire for the effect measurement by pupils comprised of 17 propositions aimed to determine the respondents’ opinions on the content (nine propositions) and teaching method (eight propositions).

Measurement was done using a 5-point Likert scale that ranged from *completely disagree* (1) to *completely agree* (5). For the content, aspects such as attractiveness, level of difficulty, and use of material were measured. The measurement of the teaching method included the way in which the teacher dealt with the teaching methods, how (s)he used the material, and linking to the initial situation.

Cronbach’s alphas for the process evaluation questionnaires were calculated. The coefficients of the different lessons ranged from 0.789 to 0.859. The internal consistency of coefficients were also checked per subject per dimension (content and teaching method). The coefficients of the dimension content ranged from 0.659 to 0.808, and those for the teaching method from 0.693 to 0.813.

Table 4 shows the design of the pre- and post-tests. The pre- and post-tests have identical content. The Cronbach’s alphas of both tests were calculated: pre-test, 0.602–0.729; post-test, 0.633–0.851.

**Table 4:** Description structure for pre- and post-test

**Question**	**Pre-test (Pr-T)**	**Question**	**Post-test (Po-T)**
1	Sum up/note 9 types (names) of drugs (Knowledge question [K])	1	Sum up/note 9 types (names) of drugs (Knowledge question [K])
2	Indicate which drugs belong to which 14 effects (K)	2	Indicate which drugs belong to which 14 effects (K)
3	Recognize 9 drugs from color photographs (K)	3	Recognize 9 drugs from color photographs (K)
4	Measuring of knowledge about drugs through 18 propositions (K)	4	Measuring of knowledge about drugs through 18 propositions (K)
5	Identify 7 aid institutions (K)	5	Identify 7 aid institutions (K)
6	Measuring of determinants* of behavior toward drugs through 25 propositions (Determinant measure)	6	Measuring of determinants* of behavior toward drugs through 25 propositions (Determinant measure)
**7**	Measuring of view on control over skills that show defensiveness (against drugs) **before** participating in the program (Skills question)	**7**	Measuring of view on control over skills that show defensiveness (against drugs) **after** participating in the program (Skills question)

*Attitude, subjective norm, observed behavior control, intention.

### Ethical consideration

Ethical clearance was obtained from the Ministry of Education (in particular, the Department of Inspection for Continuing Education at Junior level). A letter from the Ministry was sent to the participating school in which had been indicated that consent for the test deployment was granted and cooperation of the management and staff were asked for the (test-) implementation.

With regard to obtaining permission/cooperation of the staff, each participating staff member was personally approached. Parents of participating students were asked by the school for permission to take part. None of the parents has objected against their children participating in the (test-) implementation.

### Data analysis

Data were analyzed using Statistical Package for Social Sciences (SPSS) software version 19. For the process evaluation, the means, standard deviation, ANOVA, and Pearson correlation were calculated. For the effect evaluation, the pre- and post-test were administered. With questions 1, 2, 3, 4, 5, and 7 (see Table 4), two scores were possible, namely1 (correct/good) and 0 (incorrect/wrong). The total score is the number of correct answers.

The influence of the behavior determinants was measured in question 6. This question is based on the Theory of Planned Behavior (TPB). In question 6 pupils chose their answer by indicating a score (totally disagree–totally disagree) on a 5-point Likert scale. The General Repeated Measure (repeated measurement) was applied. (By using the General Linear Model (GLM) ‘repeated measures’ a variance analysis was carried out on the inter-dependent measurements. This occurs when in the same or similar (with the same level of measurement) measurements were carried out with the same subjects. In case N^nd^ measurements are performed on different groups, there is - apart from a ‘within subjects design’ also- a ‘between subjects design’).

The advantage of a within- subject design versus a between- subject design is that a within- subject design is more efficient in the sense that the sample need not be less large, while still a significant result is attained. This is because the scores of a person are associated with each other. This provides additional information, making the conclusions more reliable. In a between- subject design the averages of the condition will differ from each other because in one condition other subjects are present rather than in the other condition. In a within- subject design, this may cause no difference between the averages of the condition, because the same people are used for all conditions. The differences between the averages of condition parts of a within- subject design are therefore less subject to change. Due to the additional information of the link, the scores can be compared within a person. This makes it possible to assess whether the effect occurs consistently in all subjects (there is a small MS interaction); that consistency is additional information making the conclusions more reliable.

We next deal with the results of this measurement, and then a chi-square test is used to compare pre- and post-measurements. The statistical significance level was set at *p* < 0.05.

## Results

### Qualification of lessons by pupils

The pupils scored the lessons from 0 to 10. Except for the first Dutch lesson, there were no significant differences between classes (2A and 2B) and sexes. The average score for each lesson was 8.6 or higher, ranging from 8.6 to 9.4. A comparison of classes shows that class 2A had a range from 8.5–9.7 and class 2B from 8.0–9.3. There is no appreciable difference based on class and sex. With the boys, the social studies lesson scored lowest with 8.0 (range for boys: 4–10), while the girls’ lowest scoring lessons were social studies and biology with 8.7 (range for girls: 6–10). A comparison of the two classes shows that the first Dutch lesson significantly differed from the other lessons: *F*(2.120) = 0.5.41, *p* < 0.05.

### Pupils’ assessment of lesson content and teaching method

A comparison of the average scores and p-values with the assessment of lesson content and teaching method did not show any significant differences between sexes and classes. The average grade given by pupils showing their (positive) assessment was between 38 and 41. Based on the SD one can also conclude that male pupils (both in the full sample and at a class level) showed a greater spread in relation to the average score per lesson; however, this difference is not significant. There was significant difference between classes 2B and 2A with regard to the content of the social studies lesson.

There was no difference in class and sex with the teaching method of the lessons. The average score given by the pupils for (positive) appreciation was between 25 and 32 for the full sample, and in class 2B there was a greater dispersion in relation to the average score of the lesson. Again, this difference was not significant.

### Relationship between lesson content and teaching method

The results of the Pearson’s correlation show that (p ≥ 0.05) there is a strong positive relation between lesson content and teaching method (Table 5).

**Table 5:** Pearson’s correlation between lesson content and teaching method: full sample (N = 60)

	**Biology content**	**PE content**	**Dutch 1 content**	**Drawing content**	**Dutch 2 content**	**Social studies content**	**Dutch 3 content**
Biology	0.846**						
Method							
PE		0.887**					
Method							
Dutch 1			0.871**				
Method							
Drawing				0.768**			
Method							
Dutch 2					0.560**		
Method							
Social studies						0.761**	
Method							
Dutch 3							0.792**
Method							

**p > 0.01.

### Process evaluation by teachers

The text below describes the responses of teachers to questions concerning key components: reach, dose delivered, dose received, and fidelity. Comments on areas for improvement and lesson strengths are also included.

#### Reach

The teachers indicated that they checked attendance at every lesson (7 lessons for each class). All 31 pupils from class 2A attended 6 lessons. Only in one lesson three students were absent.

All 29 pupils from class 2B attended 6 lessons. Where in one session two pupils were absent.

Thus, over 80% of pupils per class participated in the drugs prevention program. According to registration details, a total of 60 pupils participated in both the pre- and post-test.

#### Dose delivered

The biology teacher indicated that the one of the lesson objectives, to teach the “consequences of tobacco, alcohol and marijuana use”, was not achieved due to a lack of time. The rest of the teachers stated that the objectives were reached.

The social studies teacher found that the teaching method of ‘asking questions’ after showing a DVD did not work due to a lack of time. Furthermore, he remarked that not all of the lesson phases were dealt with in enough depth. For organizational reasons, he reduced the introduction because time had been lost during preparation.

Regarding the drawing lesson (making an anti-drugs poster), the teacher found that the pupils got quite stressed trying to finish their poster within the allowed time. The teacher also had to repeat the instructions and criteria a number of times to ensure the students knew what they were doing.

When showing the Spanish-language film with English subtitles, the Dutch teacher had to explain some sections as a small number of pupils asked for clarification. The teacher commented that it is advisable to work with Dutch subtitles, or to provide a short explanation or summary of the film before, during, or immediately it is shown.

#### Dose received

The teachers reported being satisfied with the availability and use of the material. They estimated that most of the material was suitable for the level and age of the pupils, and that it was ready for use. The teachers also noted that the majority of the lessons based on a didactical model (i.e., didactical analysis). The person-to-person instruction round preceding the program was deemed sufficiently supportive. The teachers indicated that the trainer/instructor gave the trainees enough room to put forward their own views on lesson content and teaching methods. Biology and Dutch teachers thought parts of the text of the drugs information book was difficult for the pupils to understand. The physical education teacher said that the pictures helped to clearly show the effects of steroid use.

The physical education teacher also stated that both the information book and folders were used well by the pupils when executing their assignment (making an anti-drugs poster). With the Dutch 3 lesson, the film was judged to be a realistic portrayal of the consequences of drugs smuggling.

There was very little deviation from the instructions on how to use the material. All teachers indicated that they would use the material again.

#### Fidelity

All lessons were executed in accordance with the lesson plans provided. The lesson phases were dealt with in the indicated order. Prescribed material, instructions, and teaching methods in the lesson plans were largely implemented in accordance with the instructions.

#### Areas for improvement and lesson strengths

The teachers also noted areas for improvement and lesson strengths. These comments are shown in (see Table 6).

**Table 6:** Lesson strengths and areas for improvement

**Strengths**	**Areas for improvement**
**Biology 1**	**Biology 1**
Pictures are good; clearly show effects of alcohol and tobacco use and that pupils must distinguish between alcoholic and non-alcoholic drinks in the introduction	Putting up pictures takes time; many minutes get lost that way—perhaps a slide show
**Physical education**	**Physical education**
When dealing with several forms of drugs and narcotics, you could see that the pupils were shocked by the effects of drugs on the body	Have a short talk beforehand to prepare the pupils for the images
**Dutch 1**	**Dutch 1**
Presentation of the slogans by the pupils; this way they could express their creativity	Working together could be better
**Drawing**	**Drawing**
Every pupil was motivated	Executing the assignment takes time and there was too little time
Good collaboration	Not every member of the 5-member group was active
They were satisfied with the result	
**Dutch 2**	**Dutch 2**
Good focus on competition; incites them to take action to win	A prize at the end would be nice for the pupils
Thanks to the game the pupils remember the information better and can apply it	
**Social studies**	**Social studies**
Well-executed play in class 2A	Not all pupils watched the film with their full attention
Good participation when thinking up causes and effects	
**Dutch 3**	**Dutch 3**
Realistic portrayal of the swallowing of drugs and drug smuggling	Dutch subtitles preferable to English subtitles and translation without subtitles would be better
Clear information presented about effects	

### Effect evaluation results

#### Listing known drugs

In the pre- and post-test pupils were asked to list all the drugs they knew of. The variable time was found to have a significant main effect pre-test: *F*(1.56) = 5.68, *p* = 0.000; post-test *F*(1.56) = 8.81, *p* = 0.000. The average shows that the number of known drugs was higher after the intervention than before it.

Although there is no significant main effect, class 2A showed a greater knowledge of drugs than class 2B. Girls showed more progress than boys (this difference is not significant, see Table 7).

**Table 7:** Mean (and SD) for time, sex, and class per question in the pre-test and post-test

	**Time**	**SD Time**	**Time**	**SD**	**Male**	**SD Male**	**Male**	**SD Male**	**Female**	**SD Female**	**Female**	**SD Female**	**2A pre**	**SD 2A**	**2A**	**SD 2A**	**2B**	**SD 2B**	**2B**	**SD 2B**
	**Pre-test**	**Pre-test**	**Post-test**	**Post-test**	**Pre-test**	**pre-test**	**Post-test**	**Post-test**	**Pre-test**	**Pre-test**	**Post-test**	**Post-test**	**Test**	**Pre-test**	**Post-test**	**Post-test**	**Pre-test**	**Pre-test**	**Post-test**	**Post-test**
Listing known drugs	5.68***	3.7	8.81***	3.9	5.75	3.51	8.95	0.204	5.63	3.87	8.72	0.454	4.8	4.24	8.87	0.34	6.62	2.79	8.75	0.435
Coupling drugs with effects of use	1.63***	1.14	2.95***	1.33	1.41*	0.717	3.41*	1.47	1.77*	1.35	2.63*	0.882	1.61*	1.34	2.48*	1.15	1.65*	1.39	3.44*	1.35
Recognizing drugs in photographs	2.73***	1.75	5.35***	1.76	2.95	1.82	5.04	1.98	2.58	1.71	5.55	1.59	1.32***	0.54	5.67***	1.68	4.24***	1.77	5.00***	1.81
Propositions on drugs	7.88***	2.29	11.95***	3.67	7.33*	2.76	10.41*	3.91	8.25*	1.87	12.40*	3.17	8.83	1.67	11.87	3.15	6.86	2.44	11.34	4.13
Identifying aid organizations	1.38***	0.884	3.58***	1.78	1.58	1.13	3.7	1.98	1.25	0.64	3.5	1.63	1.67	1.1	3.74	1.78	1.06	0.37	3.41	1.7
Skills for defensibleness	6.15	2.47	6.61	3.78	5.33	2.64	6.45	3.62	6.69	2.22	6.72	3.93	6.87***	2.66	9.90***	1.64	5.37***	2.02	3.10***	1.58
Attitude	42.96*	8.49	46.64*	7.32	4.46	7.11	49.5	4.6	42	9.14	45.03	8.11	41.26	7.84	44.78	4.49	44.4	8.9	48.22	8.85
Intention	11.25***	5.25	19.25***	5.94	11,95	5.57	19.63	5.46	10.83	0.507	19,02	6.28	10.58**	5.21	17.03**	6.88	12.03**	5.28	21.81**	3.17
Behavior control	17.35*	3.88	21.94*	4.02	18.60*	3.1	21.39*	5.56	16.55*	4.15	21.39*	5.56	22.30*	2.65	21.16*	5.05	16.96*	3.23	22.75*	2.4
Subjective norm	18.65***	6.54	22.79***	1.96	19.13	4.96	23	1.63	18.36	7.39	22.66	2.07	20,61	4.91	22.96	1.49	16.4	7.5	22.59	2.4

*p > 0.05; **p > 0.01; ***p = 0.00.

The chi-square test indicates significant differences between the full sample with the pre-test and the full sample with the post-test, because a sizable increase was noticeable with all drugs when they were mentioned. The 10 drugs mentioned were cocaine, tobacco, alcohol, steroid, hashish, xtc, marijuana, crystal meth, and opium. With all 10 drugs, an increase can be noted with the number of drugs mentioned in the post-test (see Table 8).

**Table 8:** Full sample results for the pre-and post-tests: listing known drugs (N = 60)

**Drugs**	**TOTGR Pre-test %**	**TOTGR Post-test %**	***p*-value**	**Drugs**	**TOTGR Pre-test %**	**TOTGR Post-test %**	***p*-value**
Cocaine	83.0	100	*p* < 0.05	Hashish	63.3	100.0	*p* < 0.05
Tobacco	61.0	100	*p* < 0.05	Opium	60.0	81.7	*p* < 0.05
Alcohol	65.0	100	*p* < 0.05	XTC	65.0	100.0	*p* < 0.05
Steroids	66.7	100	*p* < 0.05	Crystal meth	65.0	100.0	*p* < 0.05

#### Coupling of drugs with effects of use

Fourteen possible drug effects from drug use were indicated. Pupils were asked to write down one drug associated with each of the 14 effects. The variables time *F(*1, 56) = 42.94, *p* = 0.000 and class *F*(1, 56) = 5.91, *p* < 0.05 were found be significant main effects in the pre- and post-test. Furthermore, it should be noted that there is a significant interaction effect between time and sex and time and class. The average score was higher in the post-test (see Table 7).

The results from the effect and drug coupling exercise showed a significant difference for nine effects when comparing the pre- and post-test results for the full sample. Five of these effects showed a positive shift in the post-test. Observed differences concern the following items: baldness and hair loss (*Chi*^*2*^ *=* 26.12; *df* = 1; *p* < 0.05)*,* red eyes (*Chi*^*2*^ *=* 6.42; *df* = 1; *p* < 0.05), male hair growth (*Chi*^*2*^ *=* 14.90*; df* = 1; *p* < 0.05), damage to mucous membranes (*Chi*^*2*^ *=* 8.29; *df* = 1; *p* < 0.05), development of anxiety (*Chi*^*2*^ *=* 15.09*; df* = 1; *p* < 0.05 0), coma (*Chi*^*2*^ *=* 8.16*; df* = 1; *p* < 0.05), headache (*Chi*^*2*^ *=* 8.64*; df = 1; p* < 0.05), cirrhosis of the liver (*Chi*^*2*^ *=* 33.41; *df* = 1; *p* < 0.05), and switching to hard drugs (*Chi*^*2*^ *=* 7.35*; df* = 1; *p* < 0.05). Four effects showed a decrease in the post-test. Three showed an increase in the post-verification, but were not significant. Two items/effects showed a significant decrease in the post-test. None of the pupils successfully coupled the effect dizziness in the pre-test.

#### Recognizing drugs in photographs

The tests also included 10 colored photos clearly showing various drugs. The pupils were asked to identify the drugs and write the drug name beneath the photos.

Both time *F*(1, 56) = 105.69, *p* = 0.000 and class *F*(1, 56) = 16.93, *p* < 0.05 were found to be main effects. A noticeable difference (increase) can be seen in the averages between the measurements taken before and after the test. The significant interaction effect between time and class shows that class 2A was slightly better at recognizing drugs in the post-test than 2B (see Table 7). Taking into account the recognition of each drug separately, a comparison of the full sample pre-test and post-test shows that there is a significant difference in time because of an increase in the recognition of nine of ten drugs depicted (see Table 9).

**Table 9:** Full sample results for pre-test and post-test: identification of drugs (N = 60)

**Recognized drugs**	**TOTGR Pre test %**	**TOTGR Post test %**	***p*-value**	**Drugs**	**TOTGR Pre test %**	**TOTGR Post test %**	***p*-value**
Steroids	100.0	83.3	*p* < 0.05	XTC	8.3	80.0	*p* < 0.05
Crack	3.3	58.3	*p* < 0.05	Crystal meth	3.3	13.3	*p* < 0.05
Hashish	1.7	48.3	*p* < 0.05	Marijuana	70.0	91.0	*p* < 0.05
Opium/poppy	23.3	70	*p* < 0.5	Cocaine	20.0	71.7	*p* < 0.05
Marijuana	10.0	16.7	*p* > 0.05	XTC	6.7	60.0	*p* < 0.05

#### Propositions on drugs

Eighteen propositions were presented with the intention to measure knowledge about drugs and drug-related items. There were significant main effects with the variables time *F*(1, 56) = 53.33, *p* = 0.000, sex *F*(1, 56) = 53.31, *p* < 0.05, and class *F*(1, 56) = 68.61, *p* = 0.000. The post-test averages were higher than the pre-test. There was also a significant interaction effect between sex and class *F*(1, 56) = 13.22, *p* = 0.000 and between time, sex and class *F*(1, 56) = 11.76, *p* = 0.000 (see Table 7).

Regarding the impact of time, classes 2A and 2B showed a positive shift in the post-test. Male and female pupils in class 2A scored better in the measurement beforehand than those in class 2B (see Tables 7 and 10).

**Table 10:** Mean (and SD) for propositions on drugs and aid organizations in the pre-test and post-tests, with a significant interaction effect of time, sex, and class

	**Male 2A**	**SD male 2A**	**Male 2A**	**SD male 2A**	**Male 2B**	**SD male 2B**	**Male 2B**	**SD male 2B**	**Female 2A**	**SD female 2A**	**Female 2A**	**SD female 2A**	**Female 2B**	**SD female 2B**	**Female 2B**	**SD female 2B**
	**Pre-test**	**Pre-test**	**Post-test**	**Post-test**	**Pre-test**	**Pre-test**	**Post-test**	**Post-test**	**Pre-test**	**Pre-test**	**Post-test**	**Post-test**	**Pre-test**	**Pre-test**	**Post-test**	**Post-test**
Propositions on drugs	8.66**	1.85	13.08**	2.35	6.00**	2.95	7.75**	3.30	8.94**	1.61	12.15**	3.57	7.47**	1.87	13.88**	2.44
Identifying aid organizations	2.16*	1.40	3.58*	1.88	1.00*	0	3.83*	2.16	1.36*	0.76	3.84*	1.77	1.11*	0.48	3.11*	1.40

*p > 0.05; **p > 0.01.

When comparing the full sample in the pre- and post-tests, the groups differ significantly with regard to 7 of the 18 propositions, namely: proposition 5, marijuana is also seen as a gateway drug (*Chi*^*2*^ *=* 3.75; *df* = 1; *p* < 0.05); proposition 9, the lungs are harmed by tar in tobacco smoke (*Chi*^*2*^ *=* 6.00; *df* = 1; *p* < 0.05); proposition 10, drugs can change, stimulate and numb one’s consciousness (*Chi*^*2*^ *=* 4.68; *df* = 1; *p* < 0.05); proposition 11, crystal meth affects the entire body (*Chi*^*2*^ *=* 8.78; *df* = 1; *p* < 0.05); proposition 15, marijuana comes from the plant Cannabis sativa (*Chi*^*2*^ *=* 5.91; *df = 1*; *p* < 0.05); proposition 17, doping is used to achieve top performances in a dishonest manner (*Chi*^*2*^ *=* 4.48; *df* = 1; *p* < 0.05); and proposition 18, pure cocaine looks like fine crystalline powder (*Chi*^*2*^ *=* 7.06; *df* = 1; *p* < 0.05).

#### Identifying aid organizations

In both the pre- and post-tests, pupils were presented with an overview of 18 institutes; students had to identify 7 as aid agencies. For time, the main effect is significant with both measurements taken before and after the test (*F*(1, 56) = 92.68, *p* = 0.000).

Pupils were better able to indicate aid organizations in the post-test. There appears to be a significant combined interaction effect between time, sex, and class (*F*(1, 56) = 4.35, *p* = 0.000). Regarding the impact of time, the boys in class 2B experienced a greater positive shift in the post-test. Furthermore, the girls in class 2A always scored better in the pre- and post-tests than those in class 2B (see Tables 7 and 10).

An analysis of the results for pupils’ recognition of aid organizations shows that the pupils differ significantly with all 10 instances and in the post-test (see Table 11).

**Table 11:** Full sample results for the pre-test and post-test: identification of aid organizations (N = 60)

**Institutions**	**Pre-test**	**Post-test**	***p*-value**	**Institutions**	**Pre-test**	**Post-test**	***p*-value**
Tabernacle of faith	13.3	55.0	*p* < 0.05	NieuweGrond	8.3	28.3	*p* < 0.05
PCS	67.0	71.0	*p* < 0.05	Victory out reach	6.7	41.7	*p* < 0.05
Kick the habit	11.7	51.7	*p* < 0.05	De Stem	13.3	35.0	*p* < 0.05
Nieuwe grond	8.3	28.3	*p* < 0.05	BAD	3.3	60.0	*p* < 0.05

### Behavior determinants

Time is a significant main effect for attitude *F*(1, 56) = 6.24, *p* = 0.000. The post-test showed that pupils now have a more negative attitude towards drugs. Furthermore, sex is also a main effect for attitude *F*(1, 56) = 4.11, *p* = 0.000. The attitude of both girls and boys showed a more positive shift in the post-test.

Girls have a much more negative attitude towards drugs in the post-program test. With regard to the subjective norm, time *F*(1, 56) = 20.63, *p* = 0.000 and class *F(*1, 56) = 5.30, *p* = 0.000 are found to be mains effects. The averages of both classes (2A and 2B) were higher in the post-test. This indicates that pupils became more sensitive towards their own appraisal of the social pressure to use drugs or not.

Looking now at the behavior determinant, observed behavior control, the variables time *F*(1, 56) = 39.5, *p* = 0.000 and sex *F*(1, 56) = 0.487, *p* = 0.000 are shown to be main effects. The averages were higher in the post-test. The variables sex and class have a significant interaction effect *F*(1, 56) = 5.05, *p* = 0.000. Although the boys showed a positive shift in the post-test, the girls showed a greater improvement. Thus, the girls were better able to control their behavior regarding drugs (use). With regard to the effect of time on class, 2A had a better initial score, but 2B showed greater improvement.

With intention there was a main effect for time *F*(1, 56) = 66.31, *p* = 0.000 and class *F*(1, 56) = 8.45, *p* = 0.000. There was an increase in the averages, which indicates that the pupils experienced a positive shift: there was an increase in the intention to show/execute behavior against drugs. Class 2B had a higher average than class 2A in both the pre- and post-tests (see Table 7).

### Skills for defensibleness

Twelve propositions were presented representing a skill that indicates defensibleness against drugs. The variable class has a main effect. Based on class *F*(1, 56) = 118.08, *p* < 0.05), a difference (increase) can be observed between the averages of the measurements before and after. Class 2A showed a greater positive shift in the post-test.

Two skills showed a significant decrease in the post-test, one remained stable and nine skills showed a positive shift (increase) (see Table 7).

## Discussion

### Process evaluation

The interviews with the teachers showed that they considered the process evaluation of the drug prevention program to accurately represent the success of the program. The teachers’ positive reactions regarding the implementation and/or adoption of the interventions are based on the following characteristics: appropriate level of complexity, can be communicated, can be included with existing curricula, observability of activities to be executed and results, possibility to adapt content through reversibility and modification, reformulating instructions and getting feedback [25-27].

Personal characteristics like attitude, subjective norm, observed behavior control, and the intention of the recipient or person delivering the intervention contribute to the experiences/opinions [28]. The factors that impact on the willingness of teachers to pass on the content of an intervention are as follows: the subjective norm, own effectiveness, interpretation of one’s job, the extent in which one feels responsible for passing on the content, and instrumentality of materials [29,30]. That is, the extent in which the material has clear instructions for its preparation, presentation, and evaluation and the extent in which the material leads pupils to be active and enthusiastic [31-33].

In the process evaluation, it was mentioned that in some instances, time constraints led to improvisation. With other health-promotion programs, times limitations have been described as bottlenecks [34-37].

With regard to dose delivered, it can be concluded that the teachers worked fairly well with the (sub) goals of the lessons and that those goals were achieved. With regard to dose received, the teachers reported being satisfied with the intervention. The material was received positively; it was largely suitable for the age and level of the pupils. With regard to its reach, the majority of pupils participated in the program.

The assessment/qualification of lessons by pupils depends on the way in which they (participants in an innovation/intervention) experience the session. Factors like a pupil’s initial level, attractiveness of the lesson content and materials, complexity of the lesson, instructional skills and expertise of the teacher, and application/use of multimedia appear to affect the performance of pupils [26,31,33,38,39]. Group dynamics processes can also affect a class’s assessment/qualification, as well as assessment by sex. Group dynamics can affect an individual’s performance, making it either better or worse [40,41].

### Effect evaluation

The differences that became apparent through the chi-square test can be explained with the taxonomy of Bloom [42] and Anderson et al. [43]. Educational psychology indicates that pupils can score considerably better with less complex cognitive activities/goals. These cognitive activities can include listing and/or recognizing drugs (reproduction of knowledge).

A comparison of the results of the pre- and post-tests showed an increase in the percentage of correct responses with the listing of drugs, recognition of drugs, and aid organizations. There is, however, no great increase in knowledge when coupling effects with the correct drugs, propositions on drugs, and mastery of skills [44,45]. It is difficult to give a precise reason for the occasional decreases in the post-test. It is possible that an overestimation by the pupils occurred in the pre-test, resulting in a more flattering picture of the situation. The post-test is possibly a more realistic representation because the pupils were confronted with accurate information at the intervention. With replication research, the execution of tasks can also be measured by means of observation.

With the measurement of the effect of time on sex and class, class influence is a feature of the group dynamics processes [40,46]. Items that are of influence in the class are, among others, the number of pupils, composition of the group, group atmosphere and the resulting social interaction, and the group objectives [33].

With regard to the impact of the pupils’ sex, such characteristics can also affect the overall school performance. There are clear differences between the performances of female and male pupils.

Females profit more form individual learning than from collaborative learning. Males profit from both to the same extent. Divergent patterns in the collaboration between females and males show a cognitive chasm between female and male pupils. There are also likely to be differences in the way they elaborate, mode of communication, and their leadership styles [47-49].

According to Veendrick, Tavecchio, and Doornenbal [50] the learning performance of boys can also be affected by the phenomenon whereby education is becoming feminized. Thus, boys go without male role models, with all its consequences. The difference in learning and performance motivation between individuals in groups/classes may play an important part. Thorpe [51] stated that boys are expected not to like school, as reinforced by comments from their parents and teachers. This has negative consequences for learning motivation and performance. Girls experience just the opposite and they do like school, thus performing better. A third explanation in the literature is a difference in learning styles. A learning style is the manner in which somebody best acquires knowledge. One person learns better, for instance, when the material is presented visually, while another learns best when the content is presented aurally [52]. Boys and girls presumably differ with regard to learning styles, and presumably those used in schools are not appropriate for boys. Van Langen and Driessen [53] have stated that boys perform well in mathematics and physics. They prefer learning rules and abstract facts by heart, and respond to episodic, factual, and detailed comments. Girls prefer open-ended tasks that are coupled with real situations. They respond more extensively to questions, putting lessons learned in a broad context.

## Conclusion

The dual purpose of “Study without Drugs” test-implementation was to determine the results of the process evaluation and whether the Proposed school-oriented drug prevention program was effective for the participating pupils when offered as a pilot.

From the results of the evaluation process can be stated that:

all classes were assessed as to be sufficient by the students. Furthermore, the students involved were positive about the content of the courses offered and the teaching methods used. Teachers had for the large part a quite positive opinion on the following aspects: reach, dose delivered, dose received, fidelity and. subject connection,

With regard to the measurement of the effect, it can be noted with caution that: participating pupils showed a (slight) increase in knowledge of drugs (that is, in the field of listing known drugs, recognizing drugs in photographs, identifying aid organizations) and their drug-resisting skills. There was no significant increase in knowledge regarding coupling of drugs with effects of use, Propositions on drugs). Furthermore, after attending the program, a number of behavior determinants—attitude, subjective norm, self-efficacy and intention—were more negative towards drugs.

Some limitations of this test implementation were: The test implementation has been done on a small scale, therefore it is desirable to note that the results cannot be generalized to other groups of students outside the group of respondents (no external generalizability).

The results (both process and impact) may become affected by aspects such as: impact test, instrumentation and maturation, previous experience concerning drug prevention programs offered outside the intervention.

The design of the study subject is pre-experimental, with no control group. Respondents were selected without randomization. Necessary adaptations must be made namely: application of randomization since this reduces the risk of selection bias. The research design should be in line with the experiment and control group in which respondents who are randomly selected are also to be included in both. Matching of the research ¬ populations should take place at the start of the study design. Furthermore, it is possible when analyzing data from a study conducted to perform “stratify or statistical control” (through multivariate regression analysis ¬) afterwards. Previously enumerated improvements may lead to increase of internal and external validity and generalizability of results.

Finally, it can be said that the developed program “Study without Drugs” - after adjustments on the basis of the recorded points for improvement/limitations observed - can be used as a starter for further development of evidence-based school drug prevention programs. A structural implementation of the program into the school curriculum could lead to (improvement/increase) enhance the quality of the lives of adolescents as well as reducing the risk that they will use drugs.

## Abbreviations

DDR: Drug demand reduction program; IM: Intervention mapping.

## Competing interests

The authors declare that they have no competing interests.

## Authors’ contributions

FI developed the program and the material, she led the analysis and interpretation, and drafted the manuscript. DV and VdW and participated in supervision of the design of the program. All authors read, revised and approved the final manuscript. All authors contributed to the design of this study.

## Pre-publication history

The pre-publication history for this paper can be accessed here:

http://www.biomedcentral.com/1471-2458/14/590/prepub
